# Combined Therapies of Modified Taiyi Miraculous Moxa Roll and Cupping for Patients with Lumbar Intervertebral Disc Herniation

**DOI:** 10.1155/2018/6754730

**Published:** 2018-03-28

**Authors:** Chunyue Cai, Yuefeng Gong, Dayong Dong, Jinbiao Xue, Xiaoting Zheng, Zhangfeng Zhong, Jialong Shao, Daguo Mi

**Affiliations:** ^1^Department of Orthopedics, Qidong Hospital of Traditional Chinese Medicine, Nantong, Jiangsu 226200, China; ^2^Shanghai Tenth People's Hospital, Qidong Branch, Shanghai 200072, China; ^3^Department of Hepatobiliary and Urology Surgery, Qidong Hospital of Traditional Chinese Medicine, Nantong, Jiangsu 226200, China; ^4^Guangdong Key Laboratory for Research and Development of Natural Drugs, Guangdong Medical University, Zhanjiang, Guangdong 524000, China; ^5^Department of Orthopedics, Nantong Hospital of Traditional Chinese Medicine, Nantong, Jiangsu 226001, China

## Abstract

Lumbar intervertebral disc herniation is a kind of syndrome caused by stimulation or pressure of nerve root and cauda equina due to intervertebral disc disorder, fibrous ring rupture, and pulpiform nucleus protrusion. Application of traditional Chinese medicine (TCM) including acupuncture therapy and cupping therapy is unique and effective treatment for lumbar intervertebral disc herniation in China. Hence, we try to investigate the combined clinical efficacy of modified Taiyi miraculous moxa roll and cupping therapy on patients with lumbar intervertebral disc herniation. Seventy patients were randomly assigned into combined treatment group (*n* = 35) and control group (*n* = 35). The treatment group received combined therapy of modified Taiyi miraculous moxa roll and cupping therapy, while control group received acupuncture therapy alone. Diagnostic criteria of TCM syndrome, Japanese Orthopedic Association (JOA) score, and simplified McGill pain questionnaire (MPQ) were used to evaluate the therapy. 11 and 13 out of 35 subjects in the combined treatment group had improvement > 75% and between 50% and 75%, respectively. The corresponding number was 2 and 22 of 35 subjects in the acupuncture group. There was significant difference in the clinical efficacy between the treatment group and control group (*P* = 0.036). The scores of JOA and MPQ detected in the patients of the two groups (*P* < 0.05) also showed statistically significant differences. Moreover, no serious adverse events occurred in the patients, who received cupping therapy or acupuncture. The combined or alone therapies can effectively improve the treatment efficacy in the patients with lumbar intervertebral disc herniation, while the combined therapies show more comparative effectiveness. Furthermore, the combined therapies are potentially safe and cost-effective and also benefit the improvement of short-term pain. Therefore, the combined therapies of the two ancient TCM deserve further clinical applications.

## 1. Introduction

Lumbar intervertebral disc herniation, also known as rupture of fibrous annulus of lumbar spine, refers to a kind of syndrome caused by stimulation or pressure of nerve root and cauda equina due to intervertebral disc disorder, fibrous ring rupture, and pulpiform nucleus protrusion [1, 2]. The syndrome is characterized by low back pain, lower limb pain and numbness, or sensory privation. This occurs in young adults between ages 20 and 40. The percentage of males is more than that of females with the illness [3]. Lumbar intervertebral disc herniation could have a big impact on patients' life and work. However, with a large number of people engaging in mental work in recent years, the morbidity of the illness is increasing due to the poor sitting posture, working and studying pressure, and lack of physical exercise [4]. According to the pathological changes, there are two major therapeutic methods including nonsurgical treatment and surgical treatment (open surgery and minimally invasive surgery) with the indications [5]. In the clinical practice, most of patients with the lumbar intervertebral disc herniation can be alleviated and cured with nonsurgical treatment, expect that a small number of patients require surgery [6].

Acupuncture, one of the nonsurgical treatments, is a commonly used method to treat lumbar intervertebral disc herniation [7]. The acupuncture can effectively improve microcirculation, rapidly eliminate the edema of nerve roots, and promote the absorption of aseptic local inflammation [8]. Along with the deep development of Chinese medicine theories and acupuncture, the patients who received the acupuncture therapy are increasing gradually [9]. Taiyi miraculous moxa roll, also known as Taiyi acupuncture, is a moxa-stick pressing moxibustion therapy modified by changing the prescription on the basis of the thunder-fire needle [10]. Cupping, nonpharmaceutical therapy, is applied by the vacuuming cups through burning inside on the selected acupoints to produce hyperemia or hemostasis. There are various kinds of cupping including flash cupping, medicinal cupping, wet cupping, retained cupping, needling cupping, and moving cupping. Application of traditional Chinese medicine (TCM) including acupuncture therapy and cupping therapy is unique and effective treatment for lumbar intervertebral disc herniation in China [11].

In our department, the patients with lumbar intervertebral disc herniation received the combined therapies of Taiyi miraculous moxa roll and cupping therapy to adjust the dredging sweat pore and remove obstruction. The curative effect observation and comparative analysis were also performed in the patients who received acupuncture therapy alone. Hence, the results are described as follows.

## 2. Materials and Methods

### 2.1. General Information

Seventy hospitalized patients with lumbar intervertebral disc herniation from January 2014 to August 2016 were selected and randomly assigned into treatment group (*n* = 35) and control group (*n* = 35). The treatment group received Taiyi miraculous moxa roll combined with cupping therapy, while control group received acupuncture therapy alone. The difference between the two groups in gender, age, disease course, and index score of pretreatment was not significant (*P* > 0.05), but was comparable (Tables 1-2).

### 2.2. Diagnostic Criteria

Patients with qi stagnation and blood stasis syndrome were selected according to* the criteria of diagnosis and therapeutic effect of diseases and syndromes in traditional Chinese medicine* (State Administration of Traditional Chinese Medicine) [12]. The patient-specific performance was described as follows: recent history of lumber sprain, principal pain in waist and legs, stabbing pain, regular pain, aggravated pain with pressing, stiffness waist, limited extension, and flexion. Diagnostics in traditional Chinese Medicine show that the patients have symptoms of dark-purple or petechial tongue and white or yellow thin fur. In palpation, doctor felt the deep uneven pulse in the patients.

### 2.3. Inclusion Criteria

The inclusion criteria were as follows: (1) meeting the diagnostic criteria of lumbar intervertebral disc herniation; (2) meeting qi stagnation and blood stasis syndrome; (3) range of ages being 20–70; (4) participating voluntarily in the clinical trial.

### 2.4. Exclusion Criteria

The exclusion criteria were as follows: (1) patients without the above diagnostic criteria; (2) patients suffering from other diseases with low back pain; (3) patients suffering from other severe primary diseases, neurosis, or psychosis; (4) patients with surgical indication; (5) pregnant or nursing women; (6) patients receiving other clinical trial.

### 2.5. Period of Treatments

Patients of treatment group received Taiyi miraculous moxa roll combined with cupping therapy that was given once every three days, a course of ten days. Patients of control group received acupuncture therapy alone which was given once every other day, a course of ten days. After two courses of treatments at the same time, the therapeutic effect, symptoms, and signs were observed and analyzed.

### 2.6. Acupoint Selection

**Figure 1:**
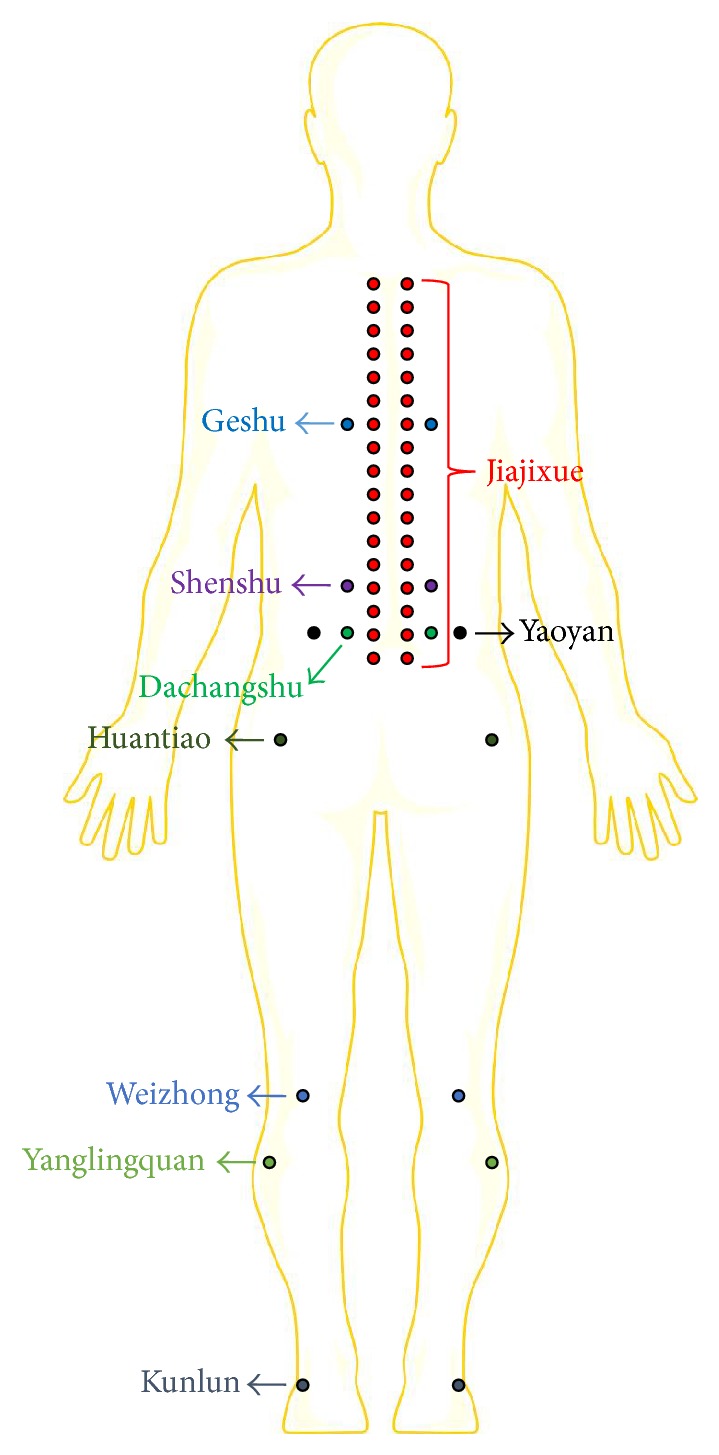
Acupoint selection in the therapy. Jiajixue, Geshu, Shenshu, Dachangshu, and Yaoyan were selected bilaterally; Huantiao, Yanglingquan, Weizhong, and Kunlun were selected from the pain side.

Jiaji acupoint is located in Jiajixue on both sides of intervertebral disc disorder according to the site of lumbar intervertebral disc herniation which was detected by CT or MRI. Other acupoints were selected according to* the treatment of diseases with acupuncture* [13], including Geshu (bilaterally), Shenshu (bilaterally), Dachangshu (bilaterally), Yaoyan (bilaterally), Huantiao (pain side), Yanglingquan (pain side), Weizhong (pain-affected side), and Kunlun (pain-affected side) as shown in Figure 1.

### 2.7. Preparation of Powder for Modified Taiyi Miraculous Moxa Roll Therapy

The prescription is composed of moxa (100 g), sulfur (6 g), frankincense (3 g), myrrh (3 g), Radix Aconiti (3 g), Radix Aconiti Kusnezoffii (3 g), pangolin (3 g), musk (3 g), realgar (3 g), asarum (3 g), cinnamon (3 g), clove (3 g), xinyi (3 g), and angelica (3 g), which were ground into powder, mixed together, and stored in a dry airtight container.

### 2.8. Preparation of Moxa Roll for Modified Taiyi Miraculous Moxa Roll Therapy

First of all, spread out a piece of mulberry squared-paper (40 cm wide). Then, spread moxa (25 g) evenly on the paper mixed with powder (6 g) and roll it tightly. Next, apply egg white on the surface and paste a piece of mulberry paper again, rolling it tightly into a moxa stick. And then, cut the moxa stick into a centimeter length of moxa rolls. At last, sprinkle a little borneol on each moxa roll.

### 2.9. Modified Taiyi Miraculous Moxa Roll Therapy

Firstly, patients lie in side or prone position with full exposure of the acupoints and pay attention to keeping warm. Secondly, location in a distance of 2.5 cm from Jiajixue on both sides of intervertebral disc disorder is the puncture point, piercing from 10 degrees outward. When there is an obvious sense of loss, stop inserting the needle. Meanwhile, when the patients could feel a radioactive tingling or an electric shock, the needle should be stopped with respect to lifting, thrusting, and rotating. Place a special hard paper on the needle handle close to the skin, insert a moxa roll into the needle handle, and light it up. In accordance with the above steps, the moxa roll is placed over the selected points, ignited, and allowed to remain onto the acupoint until it burns out completely or the patient feels a burning pain. Nip the remaining moxa roll away and replace new one to continue the therapy. Each acupoint needs burning three moxa rolls. Do the moxibustion until the skin turns red without blistering for the degree. Finally, during the moxibustion, prevent the moxa ashes from burning skin or clothes.

### 2.10. Cupping Therapy

Use flash-fire cupping at the selected acupoints. The cup retaining time at each acupoint is within 10 minutes. Use sterilized cotton to clean up the wound after removing the cup. Remind the patients to keep the wound clean to prevent infection and also to keep warm and avoid the cold [14].

### 2.11. Acupuncture Therapy Alone

Acupoint selection conducted in the acupuncture alone therapy was described as follows. After sterilizing the skin at the acupoints, use acupuncture needles (0.30 mm *∗* 40 mm) to insert Geyu (bilaterally), Shenyu (bilaterally), Dachangshu (bilaterally), Yaoyan (bilaterally), Huantiao (pain side), Yanglingquan (pain side), Weizhong (pain side), and Kunlun (pain side). The acupuncture needles were inserted swiftly and vertically into the acupoints. Adjust the depth of needle insertion according to patients' specific situation, referring to the process of the patient produces acupuncture acid, hemp, or bilge sensation. After the arrival of qi, uniform reinforcing-reducing method was performed, with retaining needle for 30 minutes, and with intermission thrice at interval.

### 2.12. JOA (Japanese Orthopedic Association) Score

JOA score was adopted to assess the curative effects by the total score of subjective symptoms, clinical signs, and daily activity limitations [15]. The range of JOA score is 0–29 points in which lower scores are associated with more serious dysfunction and worse therapeutic effect [16]. The calculation formula of recovery rate with treatment is as follows:

(1) Postoperative JOA score−Preoperative JOA score29−Preoperative JOA score×100%.

### 2.13. Simplified McGill Pain Questionnaire (MPQ)

Simplified MPQ was employed to assess subjective pain, containing pain sense (S), pain affect (A), and visual analogue scale (VAS) [17]. In detail, the total length of visual analogue scale (VAS) in MPQ is recorded as 100 mm. The score is 0 points at 0 mm, which means painless. The score is 100 points at 100 mm, which means the most severe pain [18].

### 2.14. Efficacy Evaluation Criteria

Standards for Diagnosis and Curative Effect of Chinese Medical Symptom were adopted to evaluate the efficacy with cure, improvement, and invalidity [12].

### 2.15. Statistical Analysis

All the data was analyzed by software SPSS 18.0. The measurement data was analyzed by mean ± standard deviation (*X* ± SD). The effective rate was analyzed with Pearson's chi-squared test (*χ*^2^), and *P* < 0.05 was considered as statistically significant. The Minimum Standards of Reporting Checklist contains details of the experimental design, statistics, and resources used in this study.

## 3. Results

### 3.1. Total Effective Rates

Total effective rates after alone and combined therapies are shown in Table 3. 11 and 13 out of 35 subjects in the combined treatment group had improvement >75% and between 50% and 75%, respectively. The corresponding number was 2 and 22 of 35 subjects in the acupuncture group. There were significant differences between treatment group and control group (*P* < 0.05), indicating that the combined therapies of modified Taiyi miraculous moxa roll and cupping were superior to acupuncture therapy alone referring to the total effective rates. Moreover, no serious adverse events occurred in the patients, who received cupping therapy or acupuncture.

### 3.2. JOA and McGill Scores

With alone and combined therapies, JOA and McGill scores are shown in Table 4. There were significant differences in the JOA scores between the treatment group and control group before and after therapies (*P* < 0.05), indicating that both of alone and combined therapies can alleviate the clinical symptoms and signs, but the combined therapies were superior to the alone therapy. Moreover, the scores of McGill in both groups before and after therapies were statistically significant (*P* < 0.05), indicating that both of alone and combined therapies can relieve pain. There was no significant difference in the scores of pain sense (S) and pain effect (A) between the two groups after therapies (*P* > 0.05). However, there was obvious difference in visual analogue scale (VAS) and in the McGill scores between the two groups after therapies (*P* < 0.05). Therefore, the combined therapies of modified Taiyi miraculous moxa roll and cupping were still better than acupuncture therapy.

## 4. Discussion

Lumbar intervertebral disc herniation belongs to the category of low back pain, back and leg pain, and arthralgia in TCM [6]. TCM believes that low back pain has a close relationship with the imbalance of qi and blood, meridians, and visceral functions. Limb swelling and pain may occur, as a consequence of poor circulation of qi and blood, qi stagnation, and meridian blockage [19]. Qi stagnation and blood stasis, meridian obstruction, and meridian malnutrition may lead to low back pain. Sickness due to internal injuries can be caused by liver and kidney deficiency, while exogenous pathogens can be mainly caused by kidney deficiency. Most patients with lumbar intervertebral disc herniation have some core symptoms including combined evil of inside with outside, concurrent deficiency, and excess in clinical practice. The pathogeny is summarized as follows. Kidney deficiency due to old age, lumbar dystrophy, brute force, weight lifting, evil invasion by wind-cold-dampness, and so on eventually leads to meridian obstruction and stagnation of qi and blood. The application of TCM is more important in the therapy of lumbar intervertebral disc herniation, which exhibits a unique role and curative efficacy. Acupuncture therapy, the most direct way, has the important effects in dredging meridians and moving qi and blood [20]. Modified Taiyi miraculous moxa roll is a kind of moxibustion, which has a stronger effect on dispersing cold and activating meridian, restoring qi and strengthening the immune system, and promoting and activating meridians. Cupping therapy is widely used as one of the most ancient treatment methods in China. The selected acupoints here are the back acupoints of the urinary bladder channel of Foot-Taiyang. Back-Shu points to the back of the body where the qi of the Zang-Fu organs is infused [21]. The use of cupping therapy can regulate the function of internal organs, make surface hyperemia, and improve blood circulation. Western medicine believes that the lumbar intervertebral disc herniation could be improved through stimulating by warm in the local skin. Flash-fire cupping therapy has a distinctive effect on warm stimulation. Flash-fire cupping treatment exhibits broad and strong effects, which can improve the local blood circulation resulting in regulating qi and blood, expelling the cold and dampness, activating blood and removing stasis, and warning and promoting meridians [22].

Many patients with lumbar intervertebral disc herniation try to seek the TCM therapy because they did not want to receive trauma therapy. Therefore, it is hard to get the anatomical data in TCM therapy research. In our study, modified Taiyi miraculous moxa roll was applied to the patients in the treatment group. To avoid the influence of self-limitation or fluctuated symptoms in the patients with long-term low back pain, the period of treatments was designed to be short term, 10 days, in the clinical trial. The special ingredients of Chinese herbs in the moxa roll can induce lasting and moderate effects on the acupuncture points by warm acupuncture. Otherwise, the bioactive ingredients can be absorbed by the acupuncture points resulting in a therapeutic effect. The modified Taiyi miraculous moxa roll combined with cupping therapy is based on the theory of flow blood blocking meridians and promoting without pain, which is a treatment that addresses both symptoms and root causes. The combined therapies could improve the symptoms and prevent the recurrence through promoting and activating meridians, restoring the normal function of the lumbar nerve.

## 5. Conclusion

In conclusion, combined effects of modified Taiyi miraculous moxa roll and cupping therapy on lumbar disc herniation are much more effective than that of acupuncture alone therapy in the study. Furthermore, combined therapies of modified Taiyi miraculous moxa roll and cupping are potentially safe and cost-effective. Therefore, the combined therapies of the two ancient TCM deserve further clinical applications.

## Figures and Tables

**Table 1:** Comparison of pieces of general information between two groups of patients $\left(\bar{X}\pm s\right)$.

Group	Cases	Gender		Age			Hospital stay
		Male	Female	Max	Min	Average	
Treatment	35	18	17	69	24	53.26 ± 11.64	10.03 ± 6.03
Control	35	23	12	68	25	52.77 ± 11.89	12.34 ± 6.72

**Table 2:** Comparison of JOA and MPQ between two groups before treatment ($\bar{X}\pm s$).

Group	Cases	Pain sense	JOA score	Pain effect	Visual analogue scale	McGill total score
Treatment	35	6.8 ± 2.13	13.71 ± 1.47	7.8 ± 1.21	63.49 ± 11.43	79.94 ± 10.76
Control	35	6.6 ± 2.13	13.83 ± 1.54	8.0 ± 1.40	65.40 ± 12.89	83.51 ± 12.92
*P* value	-	0.780	0.752	0.649	0.513	0.213

*Note*. The data was analyzed with *χ*^2^ test.

**Table 3:** Comparison of clinical efficacy scores between both groups after treatments (%).

Group	Cases	Excellent (improvement rate ≥ 5%)	Good (improvement rate: 50–75%)	Normal (improvement rate: 25–50%)	Bad (improvement rate ≤ 25%)	*P *(*χ*2)
Treatment	35	11	13	9	2	0.036
Control	35	2	22	9	2	(8.545)

**Table 4:** Comparison of the scores between both groups after treatments $\left(\bar{X}\pm s\right)$.

Group	Cases	Index	Before treatment	After treatment
Treatment	35	JOA	13.83 ± 1.54	23.14 ± 2.70^#*∗*^
		McGill (total)	83.51 ± 12.92	30.71 ± 6.91^#*∗*^
		Pain sense (S)	6.66 ± 2.13	3.17 ± 1.15^*∗*^
		Pain effect (A)	8.03 ± 1.40	2.71 ± 1.51^*∗*^
		Visual analogue scale (VAS)	65.40 ± 12.89	23.77 ± 6.20^#*∗*^

Control	35	JOA	13.71 ± 1.47	21.89 ± 2.25
		McGill (total)	79.94 ± 10.76	37.23 ± 6.90
		Pain sense (S)	6.80 ± 2.13	3.91 ± 1.98
		Pain effect (A)	7.89 ± 1.21	2.74 ± 1.79
		Visual analogue scale (VAS)	63.49 ± 11.43	28.80 ± 6.19

*Note*. The data was analyzed with *χ*^2^ test. ^*∗*^*P* < 0.05 versus before therapy; ^#^*P* < 0.05 versus control.

## Abbreviations

TCM: Traditional Chinese medicine
JOA: Japanese Orthopedic Association
MPQ: McGill pain questionnaire
CT: Computed tomography
MRI: Magnetic resonance imaging.
